# Ramadan Fasting and Its Impact on Patients With Chronic Kidney Disease: Insights and Guidelines

**DOI:** 10.7759/cureus.57522

**Published:** 2024-04-03

**Authors:** Sami Alobaidi

**Affiliations:** 1 Department of Medicine, College of Medicine, University of Jeddah, Jeddah, SAU

**Keywords:** nutritional considerations, clinical guidelines, renal function, chronic kidney disease (ckd), ramadan fasting

## Abstract

Fasting during the month of Ramadan is a religious practice observed by millions of Muslims worldwide, including those with chronic kidney disease (CKD). This comprehensive review aims to reflect upon the impacts of Ramadan fasting on CKD patients, excluding those on renal replacement therapy, through an analysis of clinical trials, observational studies, and expert reviews from diverse geographic and methodological backgrounds. It addresses renal function stability, broader health considerations, hydration and electrolyte balance, individual variability in fasting responses, clinical and biochemical effects, nutritional considerations, and metabolic effects. This review reveals that, with appropriate monitoring, dietary management, and individualized care plans, many CKD patients can safely participate in Ramadan fasting without adversely affecting their renal function or overall health. It emphasizes the need for a multidisciplinary approach to patient education, pre-Ramadan assessment, and post-Ramadan follow-up. Furthermore, it highlights the importance of considering individual variability and comorbidities in fasting guidance and underscores the necessity of future research to develop robust, patient-centered fasting guidelines. This review aims to provide healthcare professionals with evidence-based recommendations to support CKD patients wishing to observe Ramadan fasting, ensuring patient safety and optimizing care outcomes.

## Introduction and background

Ramadan, the ninth month of the Islamic lunar calendar, is observed by Muslims worldwide as a period of fasting from dawn to sunset. This religious practice, beyond physical abstention from food and drink, encourages spiritual reflection and increased devotion. For individuals with chronic health conditions, such as chronic kidney disease (CKD), which affects millions globally, fasting during Ramadan presents unique challenges and concerns [1].

This review article aims to provide a comprehensive overview of the impact of Ramadan fasting on CKD patients, explicitly excluding those on renal replacement therapy, such as dialysis or post-kidney transplant patients. Through consulting a broad range of recent and relevant studies, including clinical trials, observational research, and expert reviews, we present a balanced and informative perspective. The literature selected focuses on renal functions, clinical outcomes, biochemical changes, and safe fasting practices within the CKD context, drawing insights from diverse geographical locations and methodologies. Key studies by Dogan et al. [2] and Bernieh et al. [3] are instrumental in developing guidelines and recommendations for CKD patients who wish to observe Ramadan fasting.

The global prevalence of CKD, affecting approximately 700 million people worldwide, underscores the importance of understanding how Ramadan fasting impacts these patients [4]. Deciding to fast is a complex decision for individuals with CKD, involving a balance between fulfilling religious obligations and maintaining health. Despite Islamic jurisprudence allowing exemptions for those whose health may suffer, a significant portion of CKD patients, especially those not on renal replacement therapy, express a keen interest in fasting during Ramadan, viewing it as a vital part of their faith. According to Bragazzi's systematic review, up to 79% of patients with milder CKD stages wish to fast, highlighting the importance of providing these patients with comprehensive guidance and support to ensure safe fasting practices [1].

Given the notable gap in standardized guidelines and protocols for fasting among the CKD population, as highlighted by the systematic review by Bragazzi [1], the need for healthcare professionals to develop evidence-based recommendations is imperative. This ensures that CKD patients who choose to fast can do so safely, supported by informed guidance from their healthcare providers. Moreover, understanding the religious and cultural significance of Ramadan fasting is crucial for providing empathetic and effective care, highlighting the importance of bridging the gap between medical advice and patients' spiritual needs.

## Review

Effects of fasting on renal functions

Renal Function Stability

The stability of renal function during Ramadan fasting among CKD patients has been a significant focus of research, highlighting the critical need for a nuanced understanding of fasting's effects on individuals with kidney disease. A variety of studies, collectively reviewed and presented in Table 1, affirm that fasting does not detrimentally impact kidney function in CKD patients, provided that there is vigilant monitoring and informed medical guidance. This body of evidence is further supported by a retrospective propensity-matched cohort study by AlAbdan et al., who found a lower incidence of acute kidney injury (AKI) among those fasting, with an adjusted odds ratio (AOR) of 0.65. This finding suggests the potential protective benefits of fasting on renal health, reinforcing the safety of fasting for CKD patients under well-supervised conditions [5].

**Table 1:** Summary of Key Studies on Ramadan Fasting and CKD

#	Paper & Year	Population	Number of Patients	Mean GFR (mL/min) in the Fasting Group	Key Findings
1	Dogan et al. (2019) [2]	CKD Stages 3-4	79 (24 fasting, 55 non-fasting)	35.2 (29.4–42.9)	No significant alterations in renal function parameters during fasting.
2	Bernieh et al. (2010) [3]	CKD	31	29 ± 16.3	No significant impact on renal function or biochemical markers during Ramadan fasting.
3	Eldeeb et al. (2020) [6]	Hypertensive CKD patients	71 (34 with CKD and 37 without CKD)	32.65 ± 9.32	Improved peripheral and central blood pressure control, decrease in serum creatinine, and an increase in eGFR.
4	Yousef et al. (2021) [7]	CKD patients	20	25.7 ± 5.62	Fasting may improve the state of chronic inflammation without affecting body composition or deterioration of renal function tests.
5	Hassan et al. (2018) [8]	Stage 2–4 CKD	57: 31 fasting and 26 non-fasting	43.4 ± 19.1	No significant deterioration of renal functions and with a reasonable degree of safety.
6	Kara et al. (2017) [9]	Stage 3-5 CKD	94 (45 fasting and 49 non-fasting)	42.6 ± 9.8	Fasting was not associated with an increased risk of declining renal functions.
7	Ansari et al. (2022) [10]	CKD patients	28	56.1 ± 33.3	Stable early-stage CKD patients can fast with careful monitoring; however, there is a risk of renal function deterioration in advanced CKD.
8	Bakhit et al. (2017) [11]	Moderate-to-severe CKD	65	31.1 ± 13.3	Fasting during the summer months was associated with worsening renal function.
9	Chowdhury et al. (2019) [12]	Diabetic CKD Stages 3-5	139 (68 fasting, 71 non-fasting)	29 ± 16	No significant changes in clinical or biochemical parameters or adverse events.
10	Al Muhanna (1998) [13]	CKD	36	Not Available	eGFR reduction indicating possible renal deterioration during fasting.
11	El-Wakil et al. (2007) [14]	CKD	15 CKD patients and 6 healthy individuals	33.3 ± 21.1 (radio-isotope)	No significant difference in renal function but increased tubule markers in the urine of the fasting group.
12	NasrAllah et al. (2014) [15]	CKD	106 completed the study (52 fasting and 54 non-fasting)	27.7 ± 13	Fasting increased serum creatinine and heightened cardiovascular risks in those with existing heart conditions.
13	Mbarki et al. (2015) [16]	CKD	60	72.85 ± 40 (creatinine clearance)	Fasting didn't significantly affect renal function. However, the study emphasizes caution and the need for enhanced monitoring.
14	Ekinci et al. (2018) [17]	Autosomal dominant polycystic disease patients with early CKD stage	54 (23 fasting and 31 non-fasting)	86.4 ± 18.5	No significant deterioration of renal function following Ramadan fasting.
15	Alawadi et al. (2019) [18]	Diabetic CKD Stage 3	25	48.9 ± 17.5	Fasting did not result in any significant change in the biophysical and biochemical profile.
16	Abushady et al. (2019) [19]	T2DM patients with and without albuminuria	90 (30 with CKD and albuminuria, 30 with albuminuria, and 30 with no albuminuria)	63.07 ± 3.27	Ramadan fasting improved glycemic control in T2DM patients with no significant decline in kidney functions.
17	Baloglu et al. (2020) [20]	CKD Stages 2–3	117	30-60	Ramadan fasting might increase the risk of acute kidney injury (AKI) in patients with stage 2-3 CKD, particularly those with hypertension.
18	Karatas et al. (2021) [21]	Stage III-IV CKD	130 (66 non-fasting, 64 fasting)	46.2 ± 17.82	Fasting does not deteriorate kidney functions. There was a moderate improvement in kidney functions for fasting patients.
19	Megahed et al. (2023) [22]	CKD patients	63 (50 fasting and 13 non-fasting)	Not Available	Ramadan fasting does not cause a significant extra risk of renal disease progression.
20	Baynouna AlKetbi et al. (2022) [23]	CKD	306 (205 fasting and 101 non-fasting)	Not Available	Fasting was not a significant determining factor in renal function deterioration, nor did it have any significant association with adverse events.
21	İslam M. (2023) [24]	Stage 3-5 CKD	192 (189 analyzed after exclusions)	43.54 ± 11.04	Fasting did not significantly affect eGFR in patients with late-stage CKD. Improvements observed in metabolic parameters.
22	Haroon et al. (2024) [25]	Diabetic and non-diabetic CKD patients	68	38.05 ± 14.92	No worsening of renal functions in both diabetic and non-diabetic patients with stable CKD who intended to fast.

Despite these positive outcomes, it is crucial to acknowledge certain research limitations, such as small sample sizes, the observational nature of the studies, and brief follow-up durations, as outlined in Table 1. These constraints may temper the extent to which these results can be generalized across all CKD patients. Nonetheless, the consistent evidence across studies indicates that adequate precautionary measures - such as maintaining hydration outside fasting hours and managing medications carefully -can prevent fasting from leading to renal function deterioration in CKD patients. Therefore, while theoretical concerns exist about fasting's impact on CKD patients, the literature suggests that, with appropriate medical oversight and patient adherence to prescribed precautions, fasting can be a safe practice for this patient group [14,20].

Broader Health Considerations During Fasting

Beyond renal function, Ramadan fasting presents a complex interplay with broader health considerations in CKD patients, encompassing cardiovascular health, metabolic regulation, and systemic inflammation. Significant insights provided by Eldeeb et al. [6] and Yousef et al. [7] demonstrate that fasting does not adversely affect arterial stiffness in hypertensive CKD patients nor does it intensify chronic inflammation or negatively alter body composition [6,7]. These findings are critical in establishing a broader safety profile for fasting from a cardiovascular and metabolic standpoint, reinforcing the notion that, with appropriate guidance and supervision, Ramadan fasting can be safely practiced by CKD patients. This conclusion further contributes to a growing body of evidence suggesting that the physiological adjustments to fasting are well-tolerated by patients with CKD, provided they receive proper nutritional counseling and health monitoring.

Hydration and Electrolyte Balance During Fasting

Hydration and electrolyte balance are paramount for CKD patients observing Ramadan fasting, given the challenges of restricted fluid intake during daylight hours. The study by Hassan et al. [8] emphasizes the importance of maintaining adequate hydration to mitigate the risks associated with prolonged periods without fluid intake. This research underscores the necessity for CKD patients to strategically plan their fluid consumption between Iftar and Suhoor, ensuring they consume sufficient fluids to prevent dehydration and maintain electrolyte homeostasis.

Strategic hydration planning, which includes prioritizing water over caffeinated or sugary beverages and incorporating high-water-content foods in the diet, is in line with recommendations for optimal kidney health during fasting. Attention to sodium and potassium intake is also critical, as highlighted in the literature review by AlSahow et al. [26] and the update by Habas et al. [27], to prevent exacerbation of kidney function issues and ensure electrolyte levels remain balanced during the fasting period.

A recent randomized controlled trial by Tarabeiha et al. further illuminates the significance of hydration for kidney health during Ramadan, demonstrating that increased overnight fluid intake among healthy fasting individuals led to significantly lower serum creatinine and urea levels, compared to those with usual fluid intake. This study suggests that adequate hydration could counteract the dehydration effects of fasting, supporting renal function [28].

These dietary and hydration strategies are key in maintaining renal stability and preventing adverse outcomes during Ramadan. Educating fasting CKD patients on the early signs of dehydration and electrolyte imbalance is crucial. Providing clear guidelines for fluid and electrolyte management during Ramadan is an essential step in fostering a safe fasting experience.

Individual Variability in Fasting Responses

The study by Kara et al. [9] reveals significant individual variability in the renal response to Ramadan fasting among CKD patients. This variability underscores the need for a personalized approach, considering the wide spectrum of comorbidities that influence the safety and feasibility of fasting. Particularly, the approach to managing diabetes - whether it involves insulin or oral hypoglycemic medications - affects the varying risks associated with hypoglycemia. Similarly, factors such as controlled versus uncontrolled hypertension, the presence of heart failure or volume overload conditions requiring diuretics, and the patient's functional status must be evaluated. The availability of a caregiver to intervene in potential emergencies, such as hypoglycemia or fainting episodes, is also a critical safety consideration.

Healthcare professionals should thus conduct thorough pre-Ramadan assessments, incorporating these diverse factors into their advice. Tailored guidance, recognizing the nuanced impacts of fasting in the context of CKD and comorbidities, is essential for informed decision-making by the patient [10,29]. By acknowledging the complexity of individual health profiles, healthcare providers can better support CKD patients in navigating their choices regarding Ramadan fasting.

Clinical and biochemical effects

Biochemical Changes and Clinical Implications

Ramadan fasting introduces significant biochemical alterations in CKD patients. Ansari et al. [10] observed changes in electrolytes and proteinuria levels, which, while not directly indicative of renal function deterioration, are clinically significant and necessitate vigilant monitoring. These alterations can influence the overall health status of CKD patients, requiring adjustments in treatment or dietary plans during Ramadan. Complementing these findings, the LORANS study highlighted favorable metabolic shifts, including modifications in inflammation markers, amino acids, and lipoprotein subclasses [30]. Moreover, a recent study demonstrated reductions in body weight, waist circumference, BMI, and beneficial changes in blood urea and serum glutamic-oxaloacetic transaminase (GOT) levels among healthy overweight and obese males, indicating improved renal and liver health [31]. These results underscore the need for personalized management of CKD patients during Ramadan to accommodate these metabolic and health impacts.

Nutritional Considerations and Metabolic Effects

For CKD patients observing Ramadan, precise nutritional management is essential to ensure their fasting practices contribute positively to overall well-being and metabolic health. Emphasizing a diet well-balanced in protein, essential fats, and complex carbohydrates is crucial. Bakhit et al. [11] provided comprehensive dietary guidance that includes controlled intake of potassium and phosphate to manage kidney workload efficiently, ensuring adequate hydration between Iftar and Suhoor to mitigate dehydration risks, and incorporating slow-digesting foods at Suhoor for sustained energy levels and satiety. These strategies are pivotal in preventing potential nutritional deficiencies or exacerbation of kidney conditions. Tailoring nutritional advice to individual health conditions and dietary needs supports the metabolic stability and renal function of CKD patients during Ramadan, underscoring the importance of personalized dietary management. For specific dietary recommendations for CKD patients during Ramadan, see Table 2, which aligns with the insights provided by Bakhit et al. [11], emphasizing the necessity for adjustments in dietary intake to maintain health and safety during the fasting period.

**Table 2:** Dietary Recommendations for CKD Patients During Ramadan

Nutritional Component	Recommendations
Protein	Adequate intake tailored to individual needs and CKD stage, avoiding excessive consumption.
Fluids	Sufficient hydration between Iftar and Suhoor, aligning with individual fluid restriction guidelines.
Potassium	Limit high-potassium foods, mindful of foods like dates commonly consumed during Ramadan.
Phosphate	Monitor and manage phosphate intake, especially from dairy products and processed foods.
Carbohydrates	Balanced carbohydrate intake, focusing on complex carbohydrates for sustained energy.
Fats	Include healthy fats for energy and to support overall health.
Sodium	Limit sodium intake to manage blood pressure and fluid balance.
Meal timing	Suhoor should include slow-releasing energy foods; Iftar should be balanced and not overly heavy.
Snacking	Healthy snacking options between Iftar and Suhoor to maintain energy levels.

Fasting Management for Diabetic CKD Patients

Diabetic CKD patients face unique challenges during Ramadan, especially with the need for careful blood sugar management. The study by Chowdhury et al. [12] emphasizes the importance of personalized adjustments in diet and medication to maintain well-being. Managing diabetes alongside CKD requires preventing both hyperglycemia, which can worsen renal damage, and hypoglycemia, a direct health risk. It is crucial for patients to consult healthcare providers for individualized care plans that address both renal health and glycemic control. This includes adjusting medication timings and dietary strategies to ensure stable blood sugar levels during fasting hours.

Moreover, patient education on recognizing the signs of hyperglycemia and hypoglycemia is vital, as is the support from a multidisciplinary team to create a comprehensive fasting plan. Healthcare professionals should consider the psychological impacts of fasting, such as stress, anxiety, and enhanced spiritual well-being, when advising diabetic CKD patients. Guidance that respects religious practices and addresses medical needs is crucial for a safe fasting experience. Insights from studies on CKD and fasting highlight the need for holistic care that balances physical health with psychological well-being, ensuring patients receive support that encompasses all aspects of their well-being during fasting [1,14].

Safety and guidelines for CKD patients during Ramadan fasting

Ensuring the safety of CKD patients during Ramadan fasting necessitates a meticulous approach that considers individual patient conditions and adheres to specific guidelines. The cornerstone of this approach is a thorough pre-Ramadan medical assessment for all CKD patients, which should evaluate renal function, nutritional status, and overall health. This evaluation, crucial for determining the feasibility of fasting, varies in depth according to the CKD stage and is more rigorous for those with advanced disease [29].

Identifying and managing various risk factors that could impact the safety of fasting for CKD patients is essential. These risk factors include the severity of renal insufficiency, diabetes management, the use of specific medications, environmental conditions such as hot summers and long fasting hours, and others [10,12,19,24,26]. Such considerations highlight the need for personalized fasting guidance, where, for some patients, the risks may outweigh the benefits of fasting (Table 3).

**Table 3:** Risk Factors Impacting CKD Patients' Ability to Fast During Ramadan

No.	Risk Factor	Description
1	Severity of Renal Insufficiency	Higher CKD stages increase AKI risk during fasting.
2	Diabetes Status	Patients with CKD who also have poorly controlled diabetes, use large doses of insulin or secretagogues, are at risk for hypoglycaemia, or have had a recent episode of diabetic ketoacidosis (DKA) face significant risks if they choose to fast.
3	Presence of Proteinuria	Nephrotic-range proteinuria heightens fasting risks for CKD patients.
4	Hypertension	Uncontrolled hypertension complicates fasting; may be feasible under specific conditions.
5	Cardiovascular Diseases	Requires careful evaluation before fasting due to potential complications.
6	Liver Diseases	Fasting with concurrent liver disease demands thorough assessment.
7	Hot Summers and Long Fasting Hours	Increases risks of heat stress, AKI, and kidney stones.
8	Unstable Kidney Function or Acute Illness	Fasting can exacerbate conditions in patients with unstable kidney function or acute illness by impairing fluid and electrolyte management and interrupting necessary nutrition and medication, delaying recovery and worsening health.
9	Nephrolithiasis	Kidney stone history requires careful fasting management.
10	Pregnancy	CKD pregnant women are recommended not to fast due to higher risk.
11	Nutritional Status: Malnutrition	Malnutrition during fasting worsens CKD by depriving essential nutrients, leading to quicker kidney function decline and weakened immunity.
12	Use of Specific Medications and Diuretics	diuretics can significantly increase the risk of dehydration and electrolyte imbalances during fasting due to their effects on increasing urine output, necessitating careful dose management and monitoring.

To effectively navigate the diverse and significant risk factors CKD patients face during Ramadan, healthcare providers must adopt a comprehensive and nuanced management strategy. This strategy encompasses detailed assessments, continuous monitoring strategies, and individualized care plans aimed at optimizing patient care throughout the fasting period (Table 4). Importantly, the approach to fasting must be tailored to the specific stage of CKD, as outlined in Table 5, which provides detailed guidance based on the patient's condition.

**Table 4:** Comprehensive Guidelines for Healthcare Providers on Managing CKD Patients During Ramadan Fasting

Aspect	Guidelines
Pre-Fasting Assessment	Conduct a comprehensive medical evaluation, assessing renal function, nutritional status, and overall health to determine fasting safety for each patient.
Monitoring and Management	Ensure continuous monitoring of renal function and hydration status. Be vigilant for signs of dehydration or worsening renal function, adjusting medication timing and dosage as needed.
Dietary Recommendations	Emphasize a balanced diet with adequate hydration. Provide meal planning guidance to maintain fluid balance and prevent nutritional deficiencies.
Individualized Approach	Tailor advice based on the CKD stage, comorbidities, and overall health. Recognize the diversity in CKD presentations and provide personalized care.
Post-Ramadan Follow-Up	Assess any long-term effects of fasting on renal function and overall health after Ramadan. Adjust care and treatment plans based on this assessment.

**Table 5:** Stage-Specific Fasting Guidelines for CKD Patients During Ramadan

CKD Stage	Guidelines	References
Early-stage CKD (Stages 1 & 2)	Patients with minimal kidney function impairment can generally fast safely, requiring only minimal adjustments. Key considerations include maintaining proper hydration and avoiding nephrotoxic medications during fasting hours.	[10,16,17,19]
Moderate CKD (Stage 3)	Fasting remains a possibility, but a comprehensive pre-Ramadan assessment is vital to evaluate health and determine necessary adjustments. Closer monitoring and potential modifications in diet and medication are needed to ensure fasting safety.	[2,3,6,8-12,14,18,20,23-25]
Advanced CKD (Stages 4 & 5)	Typically, fasting is not recommended due to increased health risks. However, personalized decisions may allow some to fast under strict medical supervision. Diligent monitoring and management can make fasting feasible for certain individuals at this stage.	[2,3,6-9,12,22,24]

Continuous vigilance for signs of dehydration, such as dry mouth, reduced urine output, and dizziness, or worsening renal function is vital, especially for patients with advanced CKD stages, necessitating adjustments in medication timings and dosage as recommended by Chowdhury et al. [12]. Dietary management plays a critical role in ensuring the safety of fasting CKD patients. Healthcare professionals should offer guidance on maintaining a balanced diet and adequate hydration, taking into account the patient's CKD stage and making specific adjustments to limit potassium and phosphate intake in more advanced stages [11].

Given the heterogeneity in CKD presentations, a personalized approach to fasting is imperative. This approach should consider the CKD stage, comorbidities, and overall health status, emphasizing the importance of educating patients on recognizing symptoms that necessitate breaking the fast, especially in advanced CKD where the risks are heightened.

Discussion

This review has systematically explored the nuanced impact of Ramadan fasting on patients with CKD, emphasizing the criticality of personalized management strategies to ensure their safety. Through the examination of various studies, including those summarized in Table 1, we have identified that many CKD patients can indeed safely observe Ramadan fasting, provided they do not possess significant risk factors impacting their ability to fast, as outlined in Table 3.

Appropriate monitoring, counseling, and adjustments are pivotal, as demonstrated by Baynouna AlKetbi et al. [23], in mitigating adverse effects on renal function. This supports the feasibility of fasting for a subset of CKD patients who, after a comprehensive pre-fasting assessment and without significant risk factors, feel healthy and wish to fast. It is imperative to grant these patients the opportunity to fast, acknowledging their autonomy and desires, while ensuring their health is not compromised. This approach aligns with the findings from Chowdhury et al. [12] regarding the evolving landscape of remote health monitoring technologies, which offer promising avenues to enhance patient support and adherence during fasting.

Nutritional guidance, underscored by Bakhit et al. [11], remains a cornerstone of fasting safety, necessitating dynamic dietary strategies that cater to individual patient profiles and physiological needs throughout Ramadan. The integration of medical, nutritional, and psychosocial considerations into fasting decisions, highlighted by Malik et al. [29], points to a significant gap in current practices, suggesting the need for future studies to explore patient-centered models of care.

Comparing Ramadan fasting with intermittent fasting, primarily aimed at weight loss, highlights unique considerations. Intermittent fasting focuses on metabolic health and weight reduction through specific eating windows, without typically restricting water or fluid intake [29]. In contrast, Ramadan fasting is a form of dry fasting where no food or fluids, including water, are consumed from dawn to sunset, incorporating spiritual, nutritional, and psychosocial elements [11]. This dry fasting aspect demands tailored dietary strategies to ensure health and well-being, accentuating the need for patient-centered care models that acknowledge the comprehensive demands of Ramadan beyond the health or weight-centric objectives of traditional intermittent fasting.

Advising CKD patients on fasting introduces complex ethical challenges, especially when balancing religious observance with health risks. A multidisciplinary approach, incorporating ethical considerations, patient preferences, and clinical evidence, can enhance decision-making processes. This nuanced approach is critical for healthcare providers, as suggested by Ansari et al. [10] and Haroon et al. [25], to navigate the complexities of fasting advice sensitively and informatively.

Limitations and Future Research Directions

While our review sheds light on the potential for CKD patients to safely fast during Ramadan, it is imperative to acknowledge the limitations inherent in the studies reviewed. Many of these studies feature small sample sizes, lack diversity in patient populations, and are limited by their observational nature. Such limitations highlight the need for larger, more rigorous studies with diverse populations to validate these findings. Future research should focus on the long-term outcomes of fasting in CKD patients, the impact of fasting across different stages of CKD, and the development of patient-centered fasting guidelines that can be universally applied.

The absence of comprehensive guidelines specifically tailored to CKD patients during Ramadan fasting underscores a critical gap in clinical practice. Although the International Diabetes Federation (IDF) and the Diabetes and Ramadan (DAR) (IDF-DAR) Practical Guidelines provide a foundation, dedicated efforts are needed to develop robust, CKD-specific fasting guidelines, echoing the urgency highlighted by Hassanein et al. [32].

## Conclusions

In conclusion, our review emphasizes that, with appropriate monitoring and support, many CKD patients can safely observe Ramadan fasting. This highlights the need for ongoing collaboration between patients and healthcare providers to navigate this significant religious observance, ensuring that patient care and outcomes are optimized.
